# Numerical investigation of droplets in a cross-ventilated space with sitting passengers under asymptomatic virus transmission conditions

**DOI:** 10.1063/5.0070625

**Published:** 2021-12-21

**Authors:** C. Peña-Monferrer, S. Antao, R. Manson-Sawko

**Affiliations:** IBM Research Europe, The Hartree Centre, Warrington WA4 4AD, United Kingdom

## Abstract

Asymptomatic virus transmission in public transportation is a complex process that is difficult to analyze computationally and experimentally. We present a high-resolution computational study for investigating droplet dynamics under a speech-like exhalation mode. A large eddy simulation coupled with Lagrangian tracking of drops was used to model a rectangular space with sitting thermal bodies and cross-ventilated with a multislot diffuser. Release of drops from different seat positions was evaluated to analyze the decontamination performance of the ventilation system. The results showed an overall good performance, with an average of 24.1% of droplets removed through the exhaust in the first 40 s. The droplets' distribution revealed that higher concentrations were less prevalent along the center of the domain where the passengers sit. Longitudinal contamination between rows was noted, which is a negative aspect for containing the risk of infection in a given row but has the benefit of diluting the concentration of infectious droplets. Droplets from the window seat raised more vertically and invaded the space of other passengers to a lesser extent. In contrast, droplets released from the middle seat contaminated more the aisle passenger's space, indicating that downward flow from personal ventilation could move down droplets to its breathing region. Droplets released from the aisle were dragged down by the ventilation system immediately. The distance of drops to the mouth of the passengers showed that the majority passed at a relatively safe distance. However, a few of them passed at a close distance of the order of magnitude of 1 cm.

## I. INTRODUCTION

Public transportation is a key part of the effective functioning of our society today. Vehicles used for public transportation such as buses, trains, and airplanes have typically been characterized by a high density of passengers. The control of the indoor environment in these vehicles is critical for providing thermal comfort and reducing airborne disease transmission risk. This topic has been extensively investigated over the last few decades but takes on even greater relevance in the context of the current COVID-19 pandemic.^1,2^ A virus outbreak in a globalized era has also highlighted the need for a better understanding of ventilation strategies as well as the need to determine optimal mitigation measures. This is especially important for the current outbreak but also a prompt to design better the space and ventilation systems post-COVID-19.

Dynamics of droplets released from nose or mouth, for different human actions, and under mitigation measures have been investigated in depth, both experimentally^3–16^ and numerically.^17–39^ Different ventilation and airflow distribution systems were reviewed by Cao *et al.*^40^ for buildings and by Mousavi *et al.*^41^ for healthcare facilities. These references described conventional systems such as mixed ventilation, and displacement ventilation in addition to hybrid solutions, personal ventilation, or protected occupied-zone ventilation, among others. Special attention was also devoted to downward ventilation for healthcare facilities.^42–45^ The varied literature on the subject is focused on increasing the understanding of the optimal strategies for removing or analyzing the exhaled drops from different spaces.^46–65^ Despite the challenging task of reducing the spread of virus and lowering the risk of exposure, there are certain general recommendations to follow to improve the ventilation of buildings such as increasing outdoor air ventilation or improving central air filtration.^66^ The US Centers for Disease Control and Prevention (CDC) also adds the use of directional airflow as a protective ventilation concept for moving air in a clean-to-less-clean direction.^67^ This is recommended for areas where a clean environment requires a higher level of protection and/or where the less clean environment has a higher risk of containing airborne contaminants. According to the CDC, the application of this measure is easier to accomplish when the supply and exhaust points are located in a ceiling grid system.

Public transportation vehicles require a special consideration as their ventilation systems need to constantly renew the air while maintaining thermal comfort in high density spaces.^68–75^ Works reported in the literature have been mainly devoted to aircraft cabins^76^ because of the unique characteristics of these environments^77^ and the technical difficulties for providing clean air in flight conditions compared with ground transportation. However, a study of airflow and virus spread in buses and trains is equally important for controlling pollutant concentrations or mitigating the pandemic risks.^78–84^ Yang *et al.*^85^ presented a numerical study for analyzing the effects of diffusers on ventilation and spread of diseases in high-speed train cabins. Several types of diffusers located at different locations on the top part of the vehicle were investigated. Air was exhausted through the outlet located at the bottom. They concluded that the geometry and location of the diffusers investigated had significant effects on the transportation of contaminants. On the other hand, train operators following public health recommendations also respond to the current COVID-19 pandemic. For example, Ref. 86 reports that the entire volume of air is exchanged every 5–9 min depending on their train set. According to this source, no direct air flow is used inside the trains, but gentle indirect airflow is channeled up from the floor to keep passengers comfortable and further reduce the risk.

Silcott *et al.*^87^ performed in-flight and ground experiments on different aircrafts for analyzing the aerosol tracers generated by a given passenger at different rows and seats. Different combinations of exhalation modes (breathing and coughing) and the use or not of masks were investigated. This study concluded that the use of gaspers did not make a significant impact on aerosol risk. The experimental set-up used thermal blankets mainly placed on the headrest of the seats around the release point to increase thermal loading. The effect of body thermal plumes was not considered. You *et al.*^88^ performed a computational study of gaspers and identified that even supplying clean air, their use may have a negative impact and significantly influence the SARS infection risk. This was attributed to the fact that a high contaminant concentration in the region above the passenger's head could move the contaminant down to the breathing zone. Interestingly, the same study reported that statistically speaking the overall effect was neutral, meaning that there was an equal chance of either a positive or negative impact on passenger's infection risk. In a different approach, You *et al.*^89^ investigated a new ventilation system with diffusers installed on the floor under the seats and supplying clean air to passengers in the row behind. Combined with displacement ventilation, this system would remove the exhaled contaminants from the passengers to the exhaust in the ceiling. This study reported a decrease in the average exposure in the cabin compared with mixing ventilation and pure displacement ventilation systems. Finally, the German working committee particulate matter (AAF)^90^ stated that air supply from top to bottom in aircraft cabins contradicts the natural uplifting of warm particle loaded air and favors its spatial expansion, thus increasing the risk of infection. A reversal of the fresh air supply was recommended for consideration.

In general, the complexity of the matter lies in the presence of a polydisperse droplet buoyant flow that results in a stratification of droplets by size. The variability of the exhalation mode characteristics, with very different size population and exhaled airflows, makes it difficult to design an optimal solution for every case. Such solution might not always be compatible with an enhanced thermal mixing, and state-of-the-art ventilation systems may not be equally effective for both finer and larger particles. Moreover, the conclusions extracted from low density and high volume spaces might differ from those drawn from public transportation characterized by a high density of passengers and low volume spaces.

**FIG. 1.**
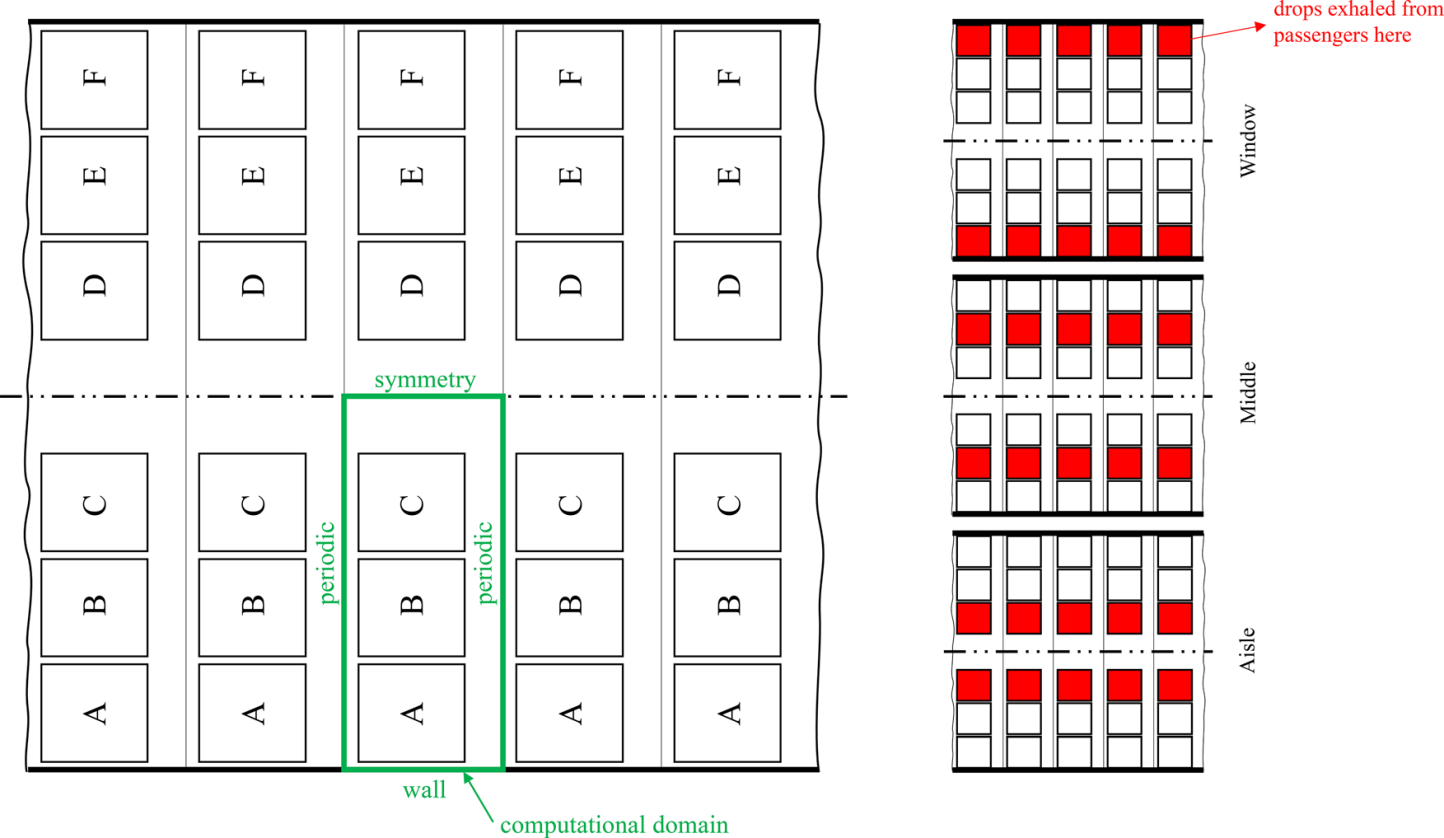
Schematic overview of the scenarios and computational domain considered.

Our work is focused on investigating speech-like modes in a public transport configuration, a scenario less investigated in the literature to date, but more characteristic of asymptomatic transmission conditions. We performed a novel Eulerian–Lagrangian simulation with high spatial and temporal resolution to understand the spread of droplets. A minimal domain was defined to achieve a high level of resolution. The domain represents a generic rectangular space with infinite rows of a 3–3 passenger layout. This is computationally modeled in this work with a row of 3 passengers using periodic and symmetry boundary conditions (BCs). Three scenarios were investigated, namely, exhalation from the aisle, middle, or window seat positions. A schematic representation of this domain is described in Fig. 1.

The simulations included transport of carrier species (air and water vapor), multicomponent particle tracking, carrier/particle vapor interaction, inflow synthetic eddy BCs for both exhalation and ventilation inlets, heat transfer, and thermal radiation. As the ventilation inlet, a multislot diffuser was placed on the side opposite the symmetry plane.

The asymptomatic virus transmission condition is represented using the approach described by Peña-Monferrer *et al.*^91^ for modeling the vocalization of vowel /ɑ/ applied to the conditions of a given female subject. This reference used experimental data to define the exhaled velocity, size distribution, and particle rate applied to a thermal manikin in a mockup. Different inlet BC approaches were evaluated considering spatial and turbulent effects at the inlet, and fluid dynamics were validated with experimental data. The conditions in Peña-Monferrer *et al.*^91^ are used in this work for reproducing a speech-like exhalation mode in a cross-ventilated space with passengers.

Mitigation strategies such as the use of masks were not considered in this work as we focus our investigation on the influence that cross-ventilation has on the spread of given pathogens carried on droplets. While future work might focus on the influence of masks within this configuration, the correct understanding of this phenomenon is of particular importance for mitigating the transmission in addition to other measures, as well as for designing new systems or operating existing ones.

## II. CFD MODELING

The computational fluid dynamics (CFD) modeling used in this work is similar to the one described by Peña-Monferrer *et al.*^91^ The reader is also referred to the extensive literature describing the methods used in this work.^92,93^ The *reactingParcelFoam* OpenFOAM v2012^94^ solver was used, which allowed us to compute in an Eulerian–Lagrangian framework, a transient, turbulent, compressible, multi-species, and multiphase particle cloud scenario. The particles were considered rigid and spherical, and rotational motion was neglected. The Eulerian part consisted of a mixture of two components, namely, air and water vapor. Turbulence was modeled using a large eddy simulation (LES) approach with a static Smagorinsky sub-grid scale model^95^ and default model coefficients.^96^ The filter length was equal to the volume-based length-scale.

The filtered governing equations for continuity [Eq. (1a)], momentum [Eq. (1b)], species transport [Eq. (1c)], and energy [Eq. (1d)] are defined as follows:

$$\frac{\partial \rho }{\partial t}+\nabla \cdot (\rho u)=0,$$ (1a)

$$\frac{\partial }{\partial t}(\rho \;u)+\nabla \cdot (\rho \;uu^{⊺})=-\nabla p+\nabla \cdot \tau +\rho \;g,$$ (1b)

$$\frac{\partial }{\partial t}(\rho \;Y_{i})+\nabla \cdot (\rho \;uY_{i})=\nabla \mu_{\text{eff}}\cdot \nabla Y_{i}+{\dot{R}}_{i},$$ (1c)

$$\begin{array}{c} \frac{\partial }{\partial t}(\rho \;h)+\nabla \cdot (\rho \;uh)+\frac{\partial }{\partial t}(\rho \;K)+\nabla \cdot (\rho \;uK)-\frac{\partial p}{\partial t}\\ =\nabla \alpha_{\text{eff}}\nabla h+{\dot{R}}_{\text{reac}},+{\dot{S}}_{\text{rad}}, \end{array}$$ (1d)

where *ρ* is the fluid density of the mixture, *t* is time, ***u*** the flow velocity field, *p* the pressure, τ the sub-grid-scale stress tensor, ***g*** the gravity vector, *Y_i_* the mass fraction of the *i*th species, $\mu_{\text{eff}}$ the effective dynamic viscosity, ${\dot{R}}_{i}$ the production rate of the given species due to reaction, *h* the specific enthalpy, *K* the kinetic energy, $\alpha_{\text{eff}}$ the effective thermal diffusivity, ${\dot{R}}_{\text{reac}}$ the heat generation by reactions, and ${\dot{S}}_{\text{rad}}$ the energy source due to thermal radiation. Reactions are neglected, and therefore ${\dot{R}}_{i}$ and ${\dot{R}}_{\text{reac}}$ are both zero for this work. The radiative heat transfer in the system for calculating ${\dot{S}}_{\text{rad}}$ is modeled using the P1 model.^97^ Finally, a one-way coupling is used, and no source term representing the particles effect in the flow is considered in the equations above.

Mixture properties are calculated from the species mass fraction and the temperature and pressure-dependent material properties of air and water vapor components are provided by a thermochemical database.^98,99^ Air is composed of ingredients with the following weights: 78.084% N_2_, 20.9476% O_2_, 0.9365% Ar, and 0.0319% CO_2_.

The Lagrangian part was formed by multiphase drop particles composed of liquid water and a non-evaporable solute phase composed of NaCl. The motion of the *j*th drop was computed by integrating Newton's second law of motion,

$$m_{j}\frac{du_{j}}{dt}=f_{j}^{g}+f_{j}^{d}+f_{j}^{l},$$ (2)

where *m_j_* stands for the *j*th drop mass, *u_j_* the instantaneous drop velocity, $f_{j}^{g}$ the gravity force, $f_{j}^{d}$ the drag force, $f_{j}^{l}$ the lift force, and *m_j_* is the total mass of the drops composed of evaporable ( $m_{e}$) and non-evaporable ( $m_{\text{ne}}$) components.

The interaction of the droplets with the carrier vapor phase was modeled as described by Chen *et al.*^100^ for calculating the mass change of the evaporable component of the drop. The material properties for the multiphase droplet were defined as liquid water with data from the thermochemical database and NaCl with values of molar mass $58.4\;\text{kg}\;{\text{kmol}}^{-1}$ and density of $2165\;\text{kg}\;m^{-3}$. If the component was totally evaporated, the remaining of the droplet with the non-evaporable component was kept tracked until they left the system through the outlet or hit a wall.

The mass fractions of water and NaCl on the drops were calculated from the volume of the sizes generated randomly from the lognormal probability density function. Sputum droplets consist of 1%–10% of their volume of solid solutes.^101,102^ de Oliveira *et al.*^27^ considered the presence of different concentrations of NaCl, proteins, and surfactants in the liquid to theoretically estimate its influence on evaporation and viral transmission. We consider in our work a dilute NaCl solution with 5% NaCl in volume for each drop without accounting for the presence of different protein concentrations or surfactants. Our work provides insights into the droplets spread in a space with cross-ventilation from a speech-like exhalation mode with a high-resolution LES. The effect that different combinations of protein and surfactants is beyond the scope of this paper.

The vocalization exhalation mode is represented with data for the pronunciation of the vowel /ɑ/ by a female. This allows us to retrieve enough information from the literature to define the glottal flow over time, particle rate, and drop particle distribution for this condition.

**FIG. 2.**
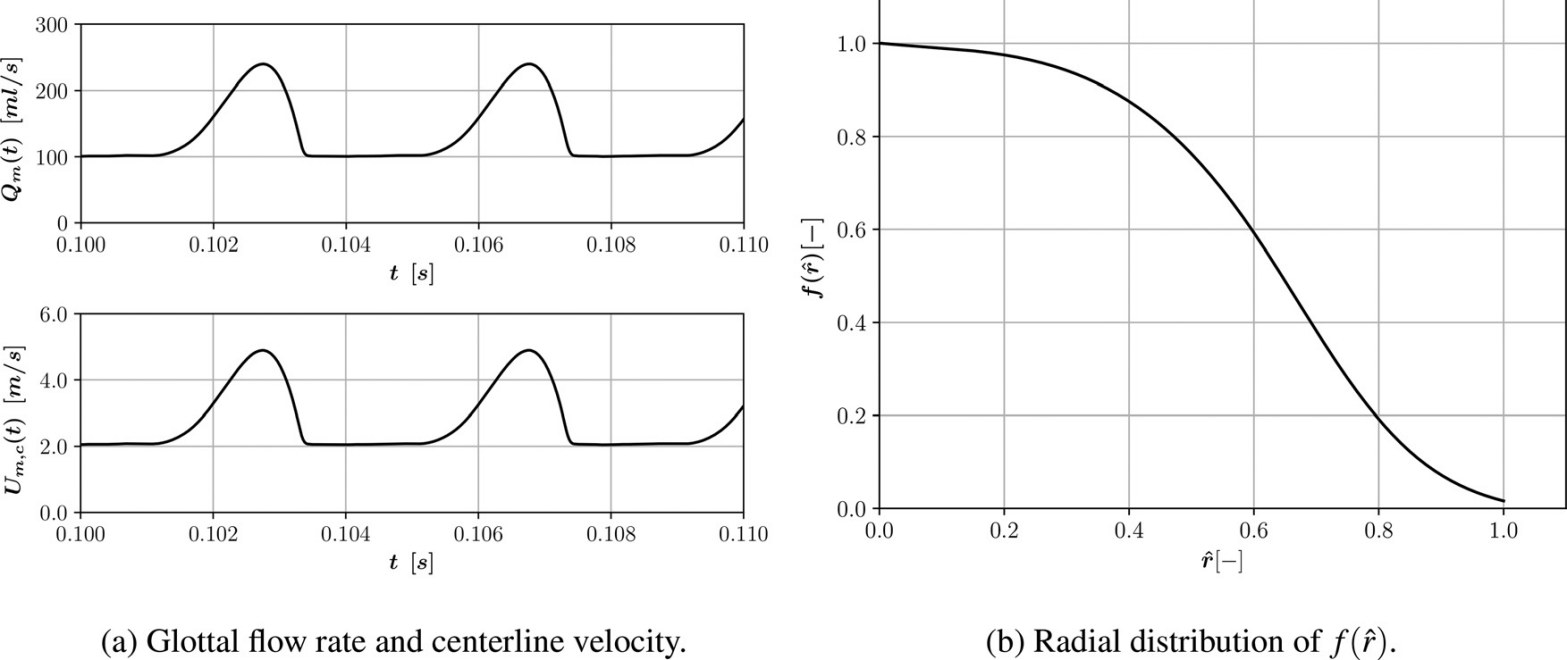
Mean velocity BC. (a) Glottal flow rate and centerline velocity as a function of time for a window of 0.01 s and (b) radial distribution of $f(\hat{r})$ as a function of the normalized mouth radius.

The airflow was calculated using the open-source repository OPENGLOT^103^ that provides a collection of data used for the evaluation of glottal inverse filtering algorithms. This repository contains synthetic data and recordings of natural vowel production and provides speech pressure signal, glottal flow, and glottal area data for different phonation and fundamental frequencies, *F*_0_. This allows us to combine data for the glottal flow with particle rate and size distribution from Asadi *et al.*^104^ for a representative female vocalizing vowel /ɑ/. The mean velocity imposed at the mouth patches over time, *t*, at any radial position, *r*, is expressed as a function of its value at the centerline,

$$U_{m}(r,t)=U_{m,c}(t)f(\hat{r}),$$ (3)

where $U_{m,c}(t)$ is the streamwise inlet velocity at the centerline and $f(\hat{r})$ is a multiplier factor function of the radial position. The latter is passed to the code as a lookup table with linear interpolation using experimental values from Feng *et al.*^105^ and using the same circular mouth opening, $r_{m}$, of 6 mm with a normalized radius of $\hat{r}=r/r_{m}$. The glottal flow waveform for the vowel /ɑ/ at *F*_0_ 250 Hz from OPENGLOT was used to define $U_{m,c}(t)$. Figure 2 shows values for the glottal flow and calculated values of the centerline velocity for a window of 0.01 s, as well as for $f(\hat{r})$.

To represent turbulent fluctuations in an LES, we use the divergence-free synthetic eddy method (DFSEM) of Poletto *et al.*^106^ for generating correlated, turbulence-like perturbations with a turbulence intensity at the mouth of 0.16. Similar to Peña-Monferrer *et al.*,^91^ we use the turbulence intensity to compute the Reynolds stresses and define the off diagonal Reynolds stress components as negligible.

The particles were seeded at the patch representing the mouth openings with a defined particle rate of 41 particles per second and a lognormal size distribution with mean 0.6951 *μ*m and standard deviation 0.7577 *μ*m. These values were obtained from the measurements by Asadi *et al.*^104^ for the fundamental frequency of *F*_0_ 250 Hz. Further details about the BCs employed for the exhalation are described by Peña-Monferrer *et al.*^91^

**FIG. 3.**
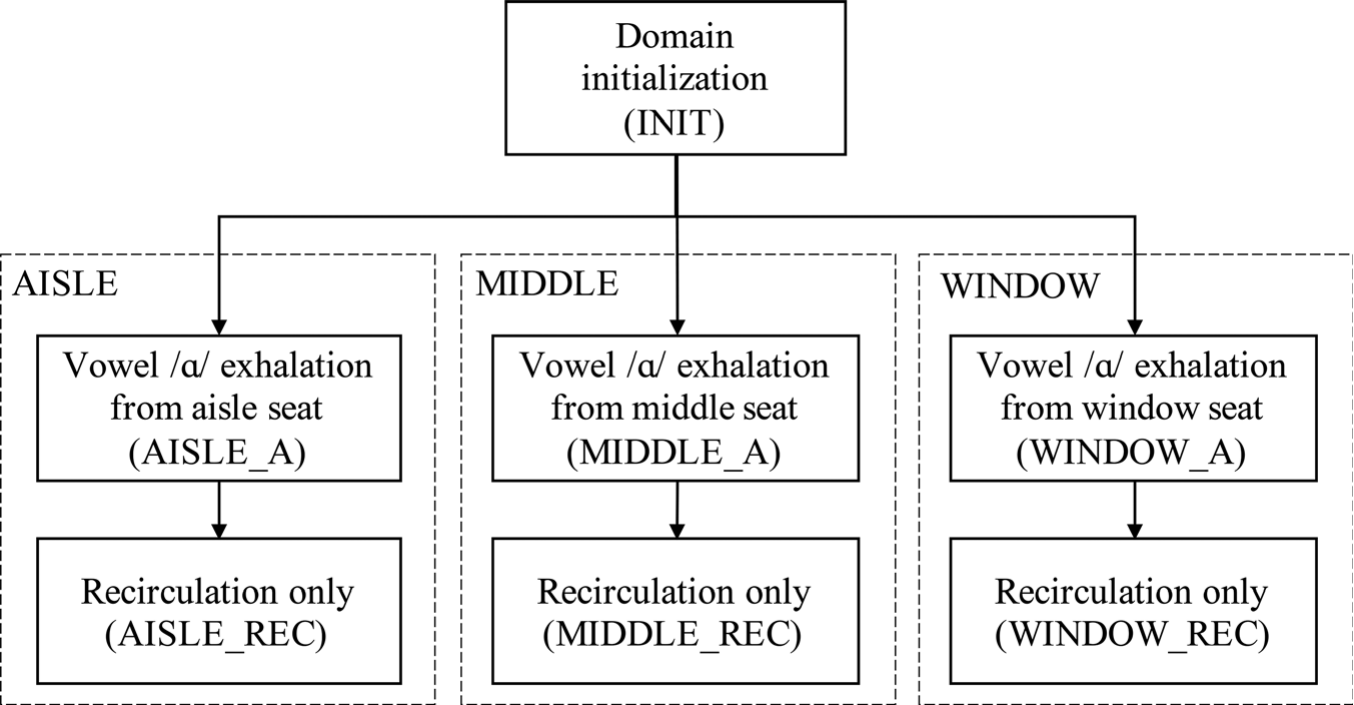
Overview and labels of the simulations.

Figure 3 shows an overview of the approach followed in this work. A first simulation for initializing the fields was performed, followed by three blocks of simulations labeled AISLE, MIDDLE, and WINDOW representing a vowel /ɑ/ exhalation for a period of 10 s (AISLE_A, MIDDLE_A, and WINDOW_A), continued by a final simulation of 30 s for observing the effect of ventilation on the distribution of droplets in the domain (AISLE_REC, MIDDLE_REC, and WINDOW_REC).

**FIG. 4.**
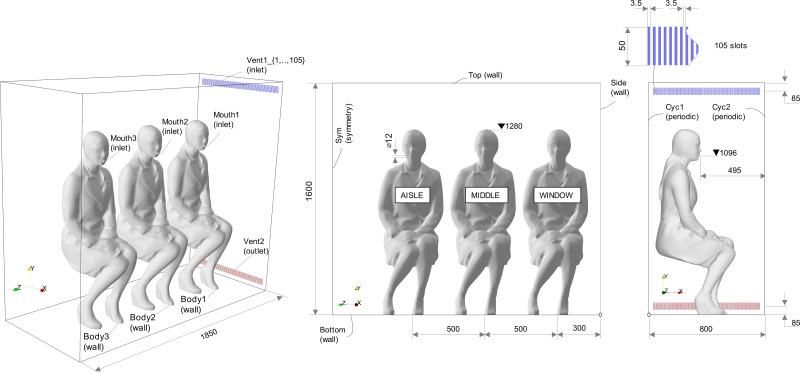
Schematic representation of the domain including dimensions and patch labels (units in mm).

The domain represents a generic space with sitting passengers in a given public transport at atmospheric pressure. A rectangular shape with infinite rows and six seats per row (3–3 layout) was considered. The computational domain of size 1.85×1.6×0.8 m (see Fig. 4) consisted of periodic BCs at the back and the front, and a symmetry plane located opposite the air injection. Particles were tracked until they hit any patch in the domain. The particles were reflected if they hit a symmetry plane. Periodic BCs were also applied to the particles on front and back patches.

The generic setup used in this work allowed us to focus on the physical phenomena independently of the specific design of external and internal features of the different public transportation vehicles. Seats and other obstacles were neglected in this study. We now discuss the consequences of using periodic and symmetry BCs. Both could result in a nonphysical settling of droplets that could slightly alter droplets' spatial distribution and the number of droplets deposited or escaping through the exhaust. The periodic BC limits the large-scale turbulent structures. In this case, the width of the computational domain was large enough to represent the longitudinal turbulent structures of the ventilation jet. The symmetry BC would affect the turbulent structures and limit the turbulent statistics around this plane in particular. However, turbulent intensity is expected to be lower in this region compared to the opposite plane where the air is injected. Symmetry encourages also a vertical downdraft which in nonsymmetric flow would likely be oscillating over a range of angles. The issues highlighted above will be more important over long time periods, but the objective of this study was to elucidate the interactions between exhalation, body plume, and cross-ventilation which happen shortly after droplet injection. The application of periodic and symmetry BCs allowed us to work with smaller domains and increase the resolution to the more turbulent regions of interest while retaining representative flow behavior in the surrounding domain. Finally, this set-up is especially useful for validating other simplified models that can later be applied to the whole domain. Similarly, this strategy can also be applied to specific full designs of buses, aircraft cabins, or trains.

The geometry of a sitting female^107^ was used to model the passengers. The vocalization of vowel /ɑ/ was also imposed for a given female subject at the mouth patches. Consecutive passengers were put at a distance of 500 mm, with the first passenger placed at 300 mm from the sidewall with the ventilation inlet. This latter passenger was considered as the one located at the window in a given transport vehicle, followed by the middle and then aisle.

A synthetic inflow BC was defined at the mouth for AISLE_A, MIDDLE_A, and WINDOW_A using the synthetic eddy method. Non-slip conditions were applied at the mouth for the rest of scenarios INIT, AISLE_REC, MIDDLE_REC, and WINDOW_REC. The current work does not consider the effect produced by inhalation, which may slightly alter the flow field near the mouth and to a different extent if masks were used. Further investigations will be focused on extending this study by including the effects of inhalation on the near mouth region and under different mitigation measures.

The flow was injected from the lateral side of the domain producing the cross ventilation, and the outlet was defined at the bottom on the same plane. The configuration of the ventilation system plays an important role in the fluid dynamics in the domain. In order to reproduce a realistic behavior at the inlet, we simulated a multislot diffuser with turbulent inlet BCs using the same synthetic eddy method as for the exhalation inflow. Every slot was defined as an individual inlet in order to apply the turbulent BC. We use the design of the diffuser described by Cao *et al.*^108^ that consisted of 105 slots on a section of length 0.8 m. This reference also provided data about turbulence intensity and velocities at the slots. In particular, we impose a supplied air to match the recommended value of 9.4 l/s per person by the ASHRAE Standard 161-2007.^77,108^ Turbulence intensity values of 35% were used based on measurements at the slot diffuser in Cao *et al.*^108^ for a similar flow rate.

Initial mass fractions in the domain for water vapor and air were defined in the simulation based on relative humidity. A relative humidity of 30% for an initial temperature of 21 °C was used. This humidity was selected to be in the boundary between an upper bound for airplanes and a lower bound for public ground transportation. Environments in flight conditions are rather dry,^109^ and standards recommended for buildings are impractical for technical reasons. Relative humidity in aircraft cabins might be as low or lower than 10%, but modern aircraft cabins target values of 25%. In contrast, trains or buses can easily reach more comfortable environments. Most people are comfortable at 30%–60% although a recommended operation range might be tighter (e.g., 45%–55%). The value of 30% is then a good compromise as a first approximation for this investigation on a generic domain. Finally, the temperature at the mouth was set to 34 °C with a 99% relative humidity.

A heat flux of $70\;W/m^{2}$ for a sedentary activity^110,111^ was considered. The effective heat loss on a real scenario depends on the type of clothing. For this reason, we differentiated between the head and rest of the body by applying $70\;W/m^{2}$ for the head on *Body1*, *Body2*, and *Body3* patches, and $20\;W/m^{2}$ for the rest of the body which is a representative value of the different clothing configurations tested in Angelova *et al.*^111^

**FIG. 5.**
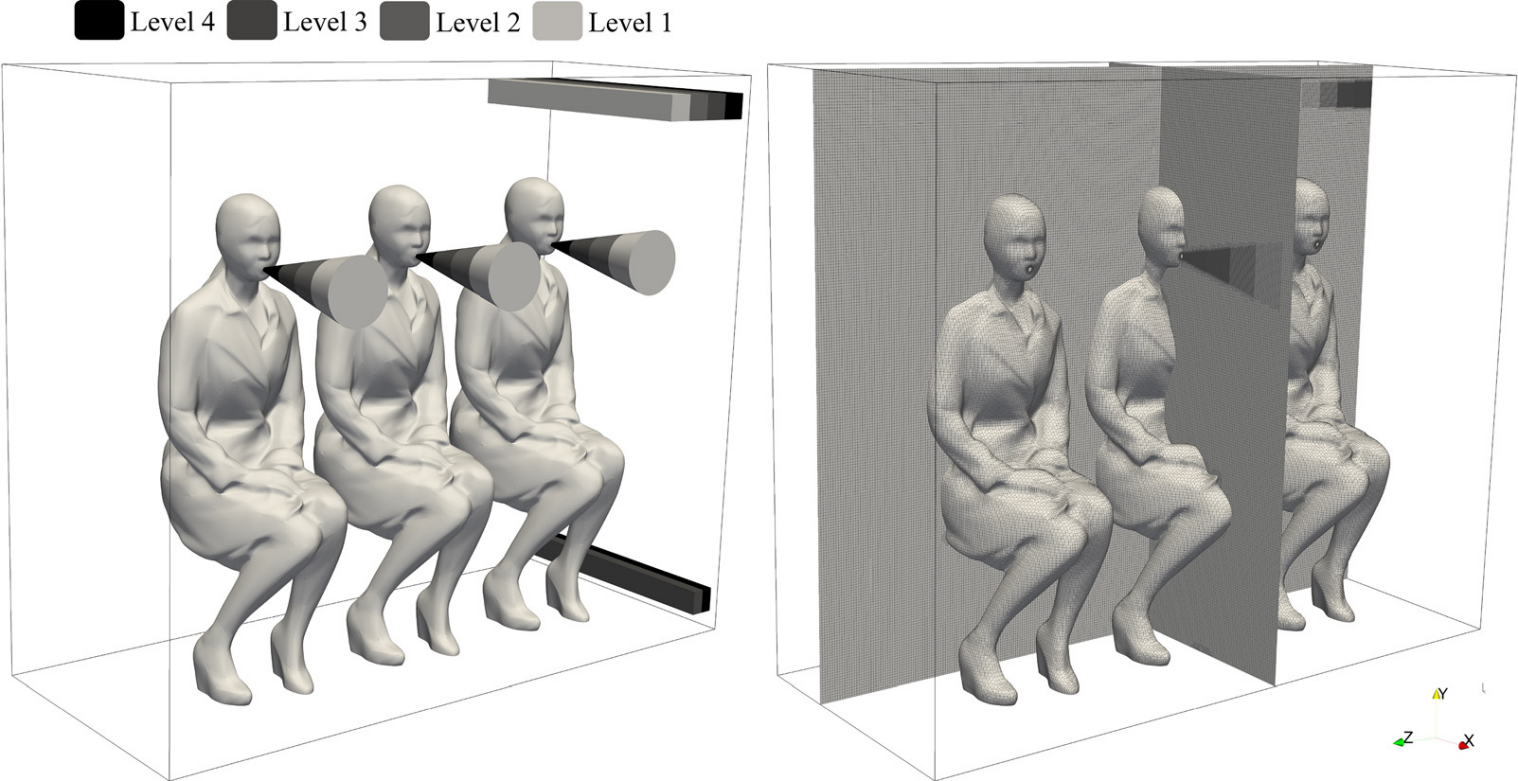
Refinement levels and mesh.

The mesh was generated using *blockMesh* and *snappyHexMesh* OpenFOAM tools, generating a mesh of 88.5 × 10^6^ elements with different levels of refinement imposed on the jet regions. This resulted in a cell size of 7 mm for the background mesh and 0.4375 mm for level 4 corresponding to the region near the mouth and ventilation inlet and outlet slots. The mesh quality parameters were within the OpenFOAM's default recommended limits with a maximum value of skewness (as defined in Jasak^112^) non-orthogonality and an aspect ratio of 3.45, 59.79°, and 5.82, respectively. Figure 5 provides details about the mesh and refinement levels.

## III. RESULTS

The simulations were executed using 440 cores per run on a high-performance computer (HPC) system. Each node on the HPC comprised two IBM^®^ POWER9^TM^^113^ processors with 22 cores each, running at 2 × 3.86 GHz, 512 GB RAM/node, and 6 NVIDIA Tesla V100 SXM2 16 GB graphical processing units (GPUs). The operation system was RHEL 7.6. An in-house GPU accelerated version was developed and used for these simulations. The sparse approximate inverse preconditioner^114^ for the pressure equation was adapted from the RapidCFD implementation^115^ and the scalar-field solver was reimplemented in CUDA. We leveraged the neighboring of ranks to expose asynchronous execution opportunities to reduce latencies at scale using the NVIDIA Collective Communication Library (NCCL). The speedup achieved was 2.3× for this case. Note that this implementation performs better, but the calculation is the same as in standard OpenFOAM.

The simulation was run with an adaptive time step based on a maximum Courant number of 1 that resulted in an average time step of 0.13 ms. An overview of the physical simulation time and computational wall clock time for each of the simulations is shown in Table I.

**TABLE I.** Overview of physical simulation time and computational wall time.

Label	Physical time (s)	Wall time (h)
INIT	200	1576
AISLE_A	10	101
MIDDLE_A	10	104
WINDOW_A	10	106
AISLE_REC	30	249
MIDDLE_REC	30	250
WINDOW_REC	30	258

**FIG. 6.**
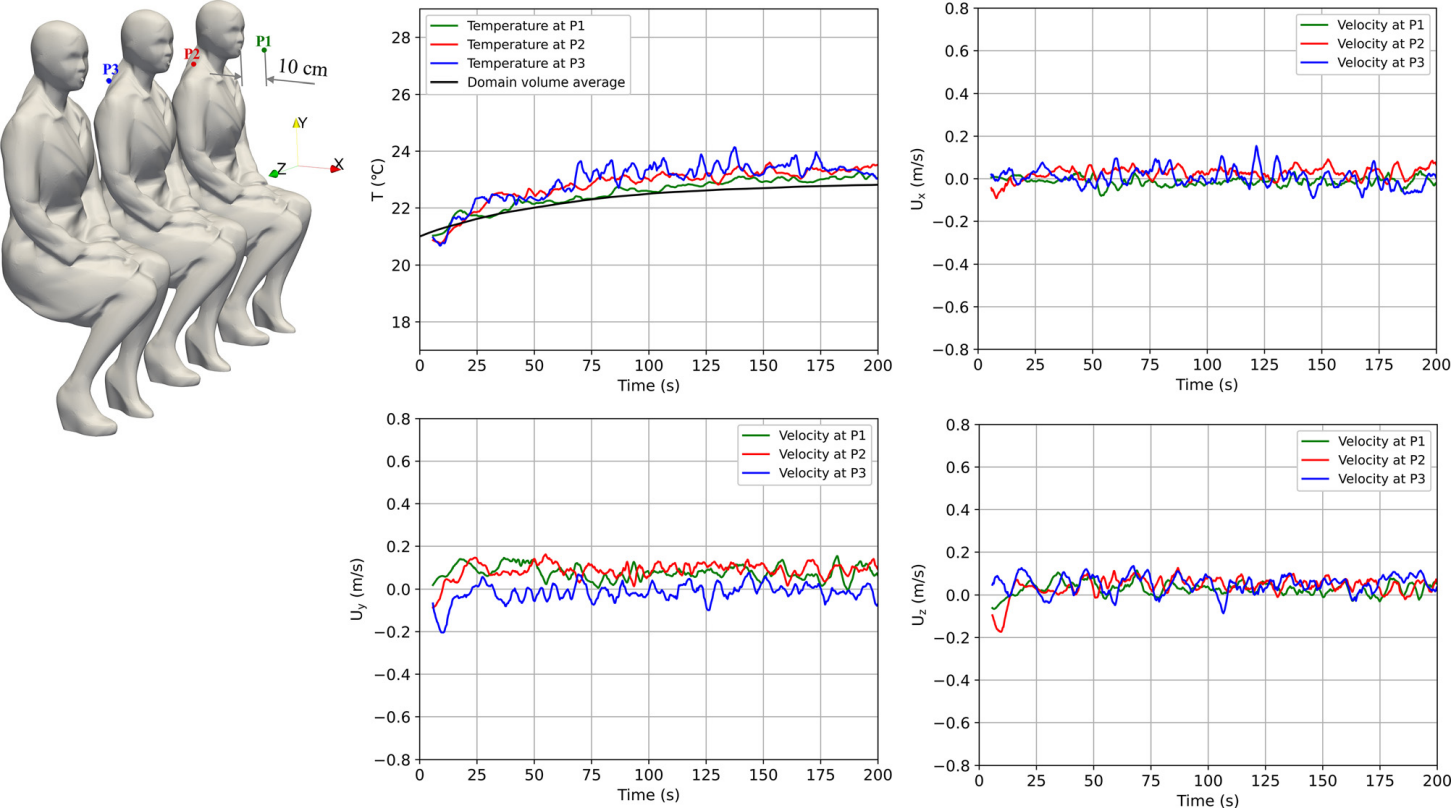
Evolution of temperature and velocity components at monitoring points for INIT.

The INIT simulation was run for 200 s until values of temperature and velocity were statistically stable. Three monitoring points were placed at 10 cm from the mouth of each passenger (P1, P2, and P3). Figure 6 shows temperature and velocity evolution over time at these monitoring points together with volume-averaged values of temperature.

Simulations considering the vocalization experiment for the three different passenger positions were run for 10 s starting from INIT, followed by the 30 s of only recirculation. The results shown hereon include the combined results for the total time of 40 s with labels AISLE, MIDDLE, and WINDOW scenarios.

**FIG. 7.**
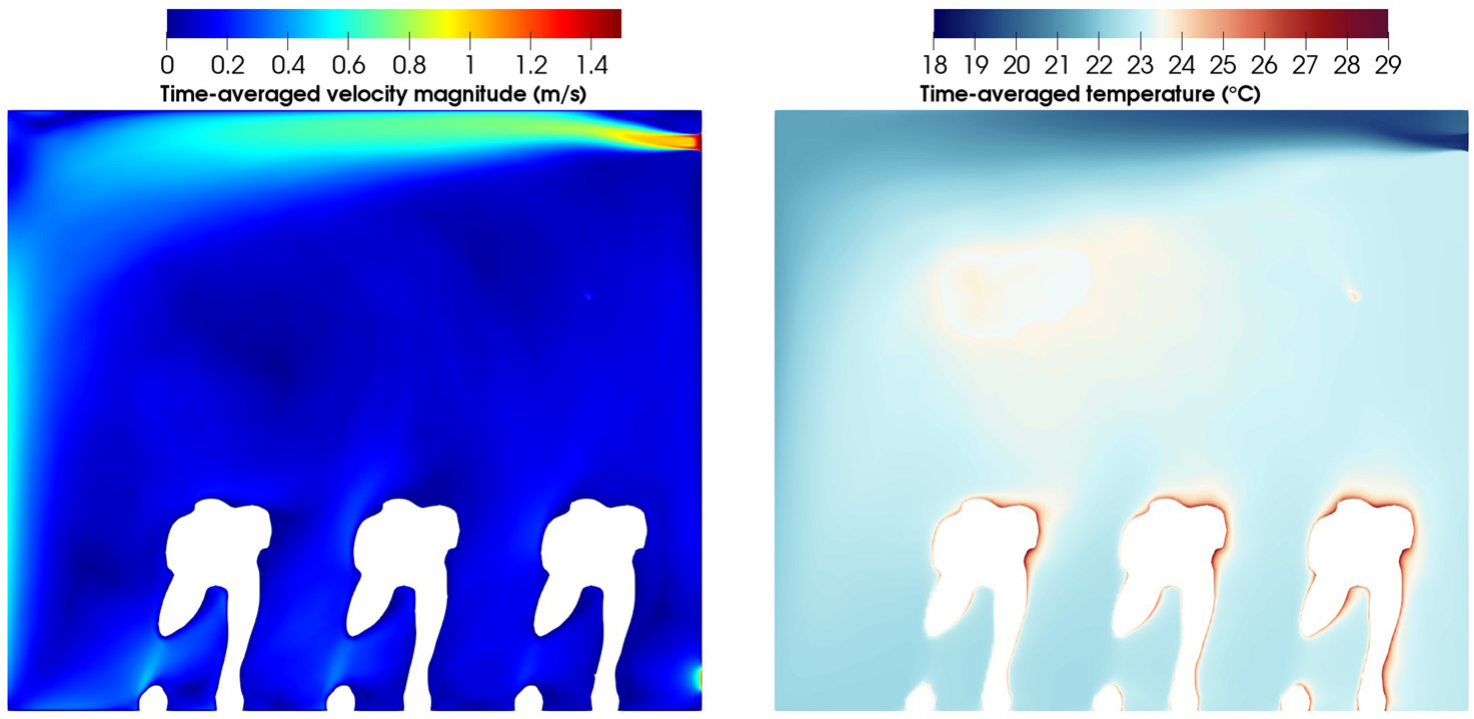
Time-averaged velocity magnitude (left) and temperature (right) for the WINDOW scenario at x 0.456 m.

Figure 7 describes the average velocity magnitude and temperature for the WINDOW scenario. The velocity field shows the primary and secondary recirculations generated in the domain. Related to the air injection, it can be appreciated how the jet attached to the top surface after the injection, presumably because of the Coandă effect. Temperature distributions show the effect that the ventilation system has on the domain with the injected cold air removing the heat generated by the passengers.

**FIG. 8.**
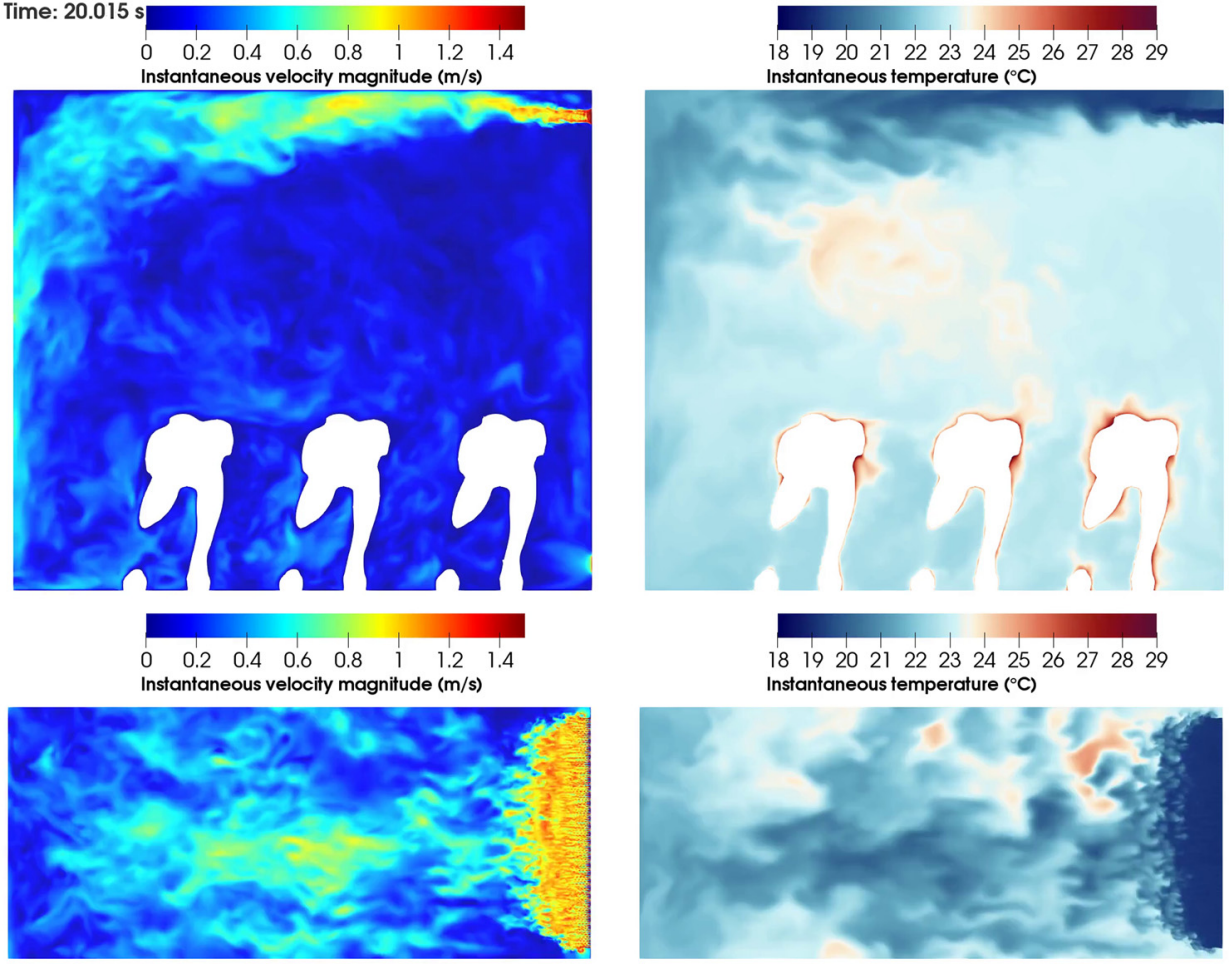
Instantaneous velocity magnitude and temperature for front (x = 0.456 m) and top (y = 1.515 m) views for the WINDOW scenario. Multimedia view: https://doi.org/10.1063/5.0070625.1
10.1063/5.0070625.1

The complete sequence of the instantaneous values for velocity magnitude and temperature is shown in Fig. 8 (Multimedia view) for the front and top views. These images give a visual representation of how droplets may interact with the fluid structures and thus give us a first glimpse of how droplets released from one passenger may affect other occupants. The top view reveals the presence of secondary flows that are generated by both velocity fluctuations at the inlet in the longitudinal direction and interaction of the fluid flows close to the symmetry plane. Although we are using half of the domain in the transverse direction, the simulation accounts for the effect of two jets encountering at the symmetry plane in this domain.

**FIG. 9.**
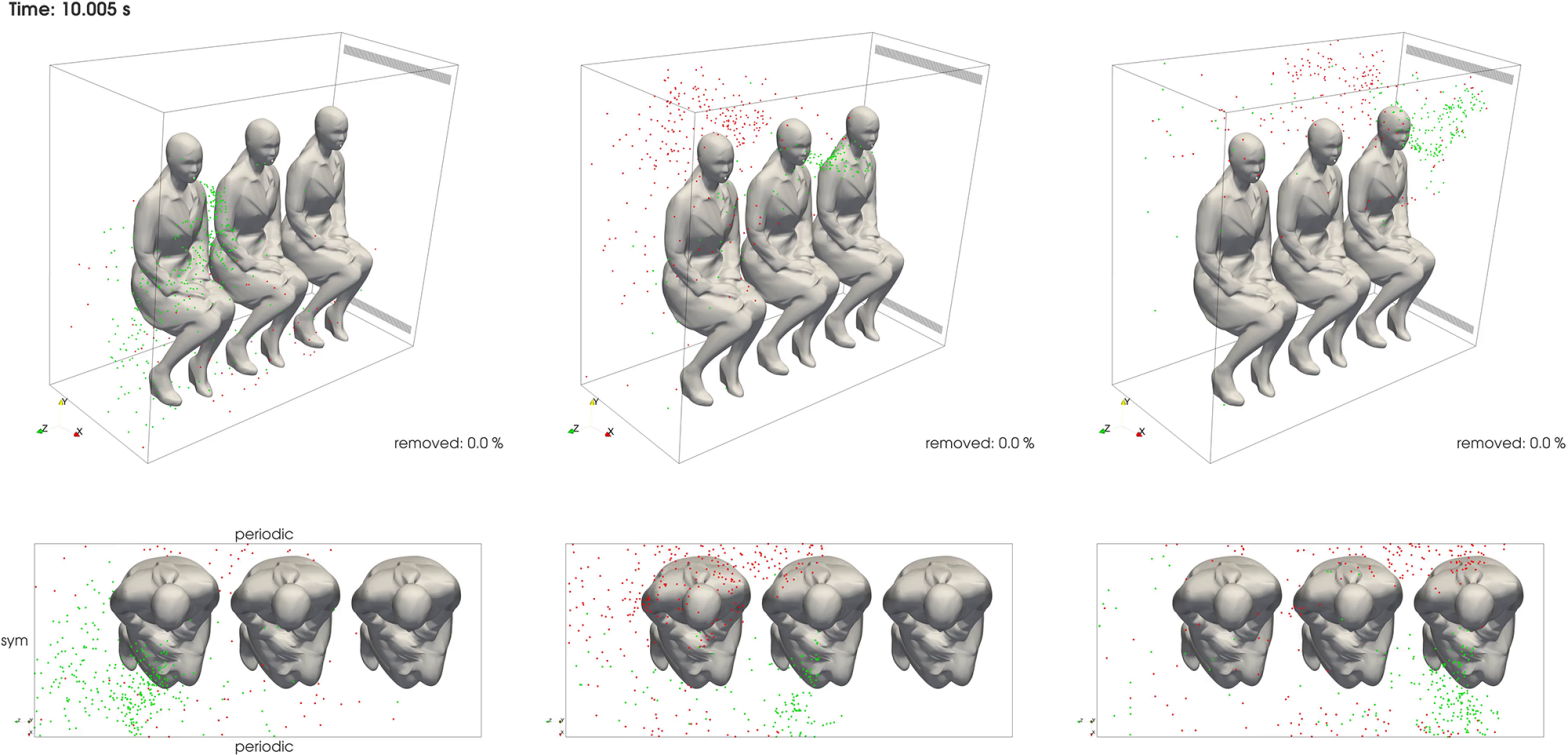
Droplets evolution for AISLE, MIDDLE, and WINDOW scenarios for 3D and top views. The images include droplets represented as spheres with the same constant radius for visualization purposes. Multimedia view: https://doi.org/10.1063/5.0070625.2
10.1063/5.0070625.2

The visualization of the droplet dynamics for AISLE, MIDDLE, and WINDOW is shown in Fig. 9 (Multimedia view). This reveals different effects such as transverse and longitudinal contamination as a result of the exhaled jet and the ventilation system effects. It provides a good comparison to identify the difference on droplet dynamics for the different seat positions. Drops are initially displayed in green and changed to red once they crossed any of the periodic BCs. This gives an idea of the dynamics of the contamination of the environments between different rows. The interaction over time of the exhalation plume and the ventilation is clearly shown in the three dimensional space. MIDDLE and WINDOW scenarios exhibited an initial plume that passed the front periodic BC representing a contamination on the back of an hypothetical front row. As the plume evolved, many of the droplets reached higher vertical positions by being dragged by the ventilation jet. This phenomenon prevented, for this condition, the core of the plume reaching the mouth or nose region of the front row during the first few seconds. This is especially important since the particle concentration is particularly high during the early stages of the exhalation process. Later, the drops were clearly affected by the secondary flows generated in the longitudinal direction, and mixing the drops as shown in the top view.

**FIG. 10.**
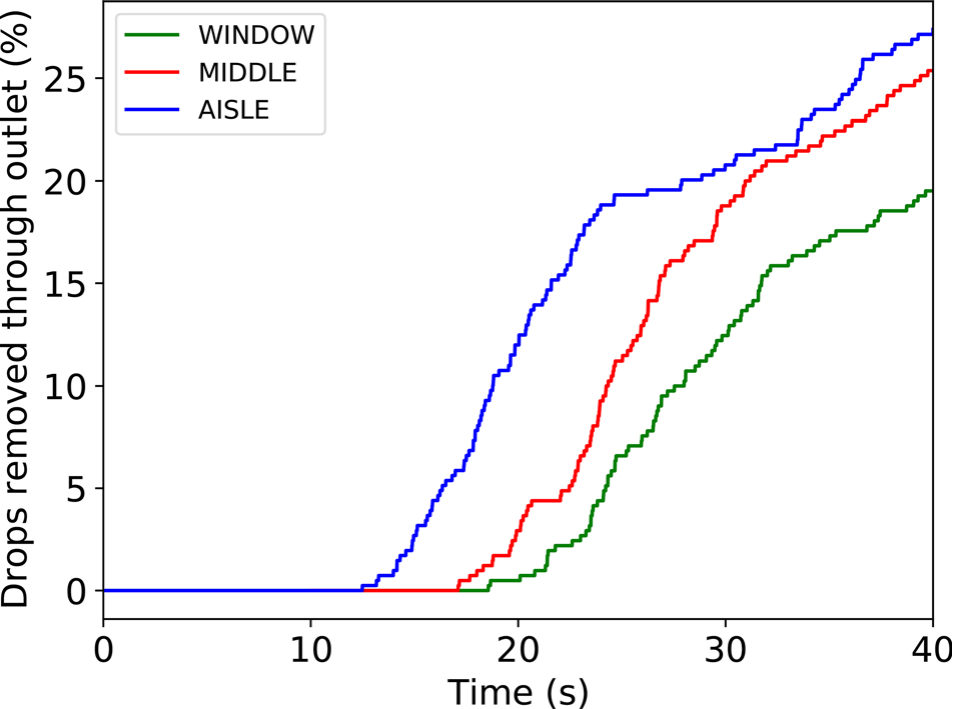
Time evolution of percentage of drops leaving the system through the outlet for AISLE, MIDDLE, and WINDOW scenarios.

A total of 27.4%, 25.4%, and 19.5% of droplets were removed through the outlet during the 40 s of simulation for AISLE, MIDDLE, and WINDOW, respectively. The evolution of droplets removed from the system through the outlet over time is shown in Fig. 10. The droplets started to leave the domain earlier for AISLE as they were more advanced on the recirculation cycle. Particles that were not removed in the first instance were driven by buoyancy and entered in a new cycle of the recirculation. After this, the particles were removed at a slower rate as they were more dispersed.

An overview of the percentage of droplets hitting a given patch is summarized in Table II. The data showed that between 13.9 and 16.1% of the droplets deposited in the passengers' bodies, top, bottom, and side walls (see Fig. 4 for the definition of the patch labels). No droplet impacted the passenger's mouth, and some relatively small number of droplets impacted the bodies. The body near the aisle (Body3) had a higher contribution for all scenarios, mainly caused by its position with respect to the primary recirculation flow. Our simulations only contained the passengers' bodies to reproduce the thermal body plumes. Other internals were deliberately neglected in this work to focus the investigation on the effect that cross-ventilation had on the evolution of droplets from the source, without specific seat designs influencing the results. The presence of seats under-seat luggage or other obstacles would increase the number of drops deposited in surfaces. The results in this paper are then more representative of spaces where the internal obstacles occupy less volume, which is frequent in transport vehicles (e.g., thin seats and frames in airplanes, or headrest less hanging seats in some trains). Ultimately, neglecting internal obstacles is likely to be the worst-scenario to consider for designing a ventilation system and reducing aerosols in the air. However, further considerations need to be made in future studies for accounting for the effect of internals in aerosols and evaluating the potential risks via indirect contact route of transmission.

**TABLE II.** Percentage of droplets hitting mouth and wall patches for AISLE, MIDDLE, and WINDOW scenarios.

Label	Mouth1	Mouth2	Mouth3	Body1	Body2	Body3	Side	Top	Bottom	Total
AISLE	0	0	0	0.7	0.5	1.5	5.6	1.5	4.2	14.0
MIDDLE	0	0	0	0.5	0.2	1.7	6.1	2.4	5.1	16.0
WINDOW	0	0	0	0.5	0.2	1.2	4.6	2.9	5.4	14.8

**FIG. 11.**
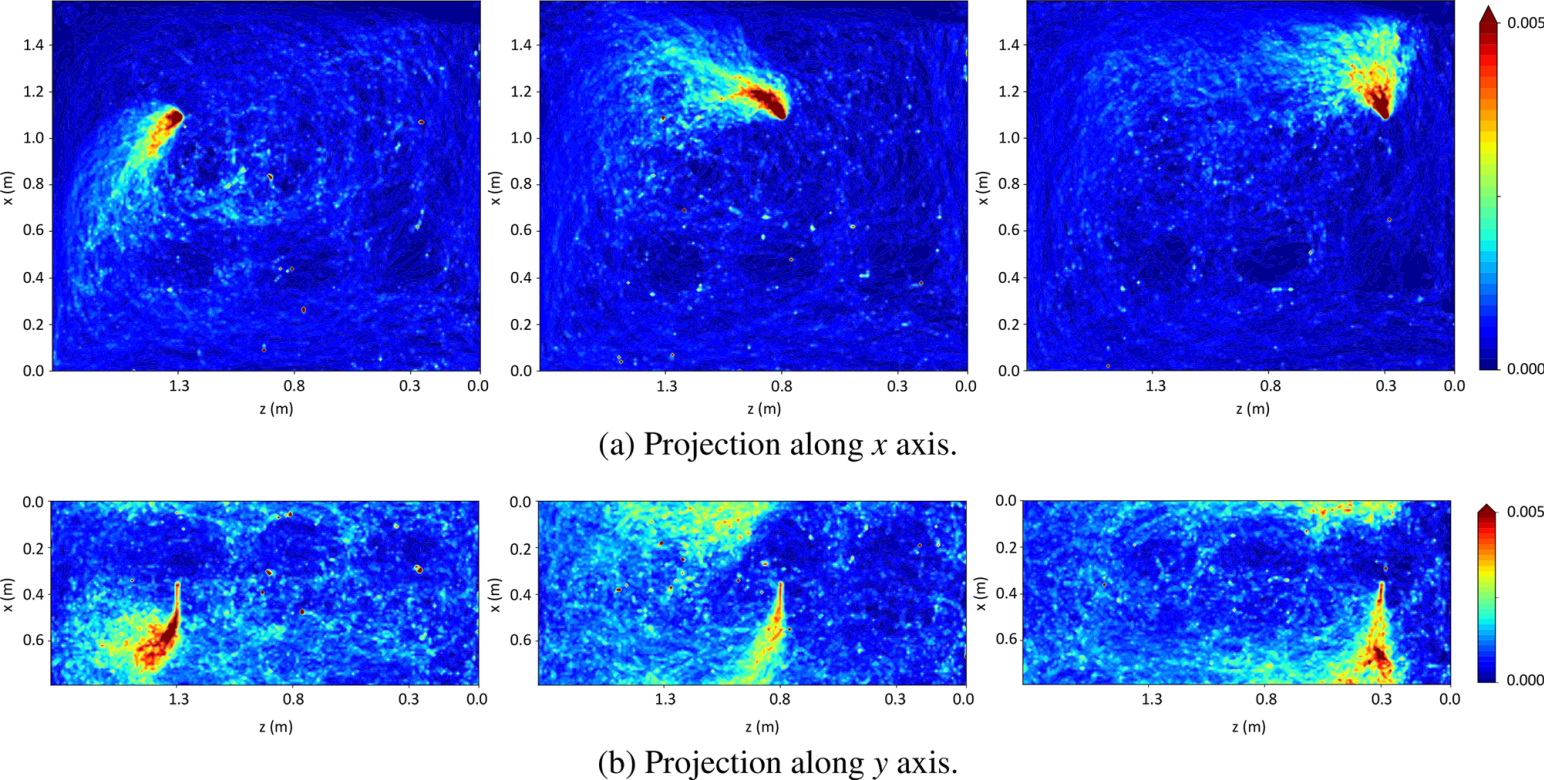
Normalized probability for particles projected on a plane along x axis (a) and y axis (b) for AISLE (left), MIDDLE (center), and WINDOW (right).

Figure 11 quantifies the spatial distribution of droplets calculated as a normalized probability of particles projected on planes along *x* [Fig. 11(a)] and *y* axes [Fig. 11(b)]. This captures several effects such as the longitudinal and transverse contamination of the environment for each case. Figure 11(b) indicates that the higher concentrations were in general less predominant along the center of the domain where the passengers sit. This is in part due to the exhaled jet momentum, which is significantly lower compared with sneezing or coughing. The results also revealed different patterns for the three different scenarios. Droplets released from the window seat raised more vertically and invaded the space of the rest of the passengers to a lesser extent. In contrast, droplets released from the middle seat contaminated the aisle passenger space as depicted in both projections. Droplets released from the aisle seat were dragged down by the ventilation system from the beginning of the event, as also visualized in Fig. 9.

Longitudinal contamination is particularly bad for containing the risk of infection to a given row, but it might have an overall positive impact by reducing local concentrations. In this vein, rapid mixing and dilution were also reported in the experiments performed by Silcott *et al.*^87^ The final positive or negative impact of this phenomenon will depend on the specific scenario and the given passenger distribution inside the vehicle. It is also worth noting that the particle sizes in the system were relatively low compared with other exhalation modes such as coughing or sneezing. Therefore, the potential amount of virus carried within the drops will also be smaller for a single event. However, speaking events are more frequent than other exhalation events taking place in symptomatic individuals. This could result in an overall higher number of virus particles in the system for a given period. Finally, we considered an infinite row of passengers without masks that represent a release of droplets from different rows at the same time, which is a more contaminated scenario than the one found in public transportation when mitigation measures are in place.

An important aspect to analyze based on these results is the effect that ventilation strategies may have on the risk of infection. Some public transportation vehicles are equipped with personal ventilation systems to give personal comfort control, which act as a downward air supply above the passengers. Our results in Figs. 9 and 11 seem to indicate that a downward flow from the passenger seat in the MIDDLE scenario might be more likely to have a negative effect for that passenger. In this case, droplets from the middle passenger that would move above the top head of the aisle passenger will be moved down to the breathing region of that passenger.

**FIG. 12.**
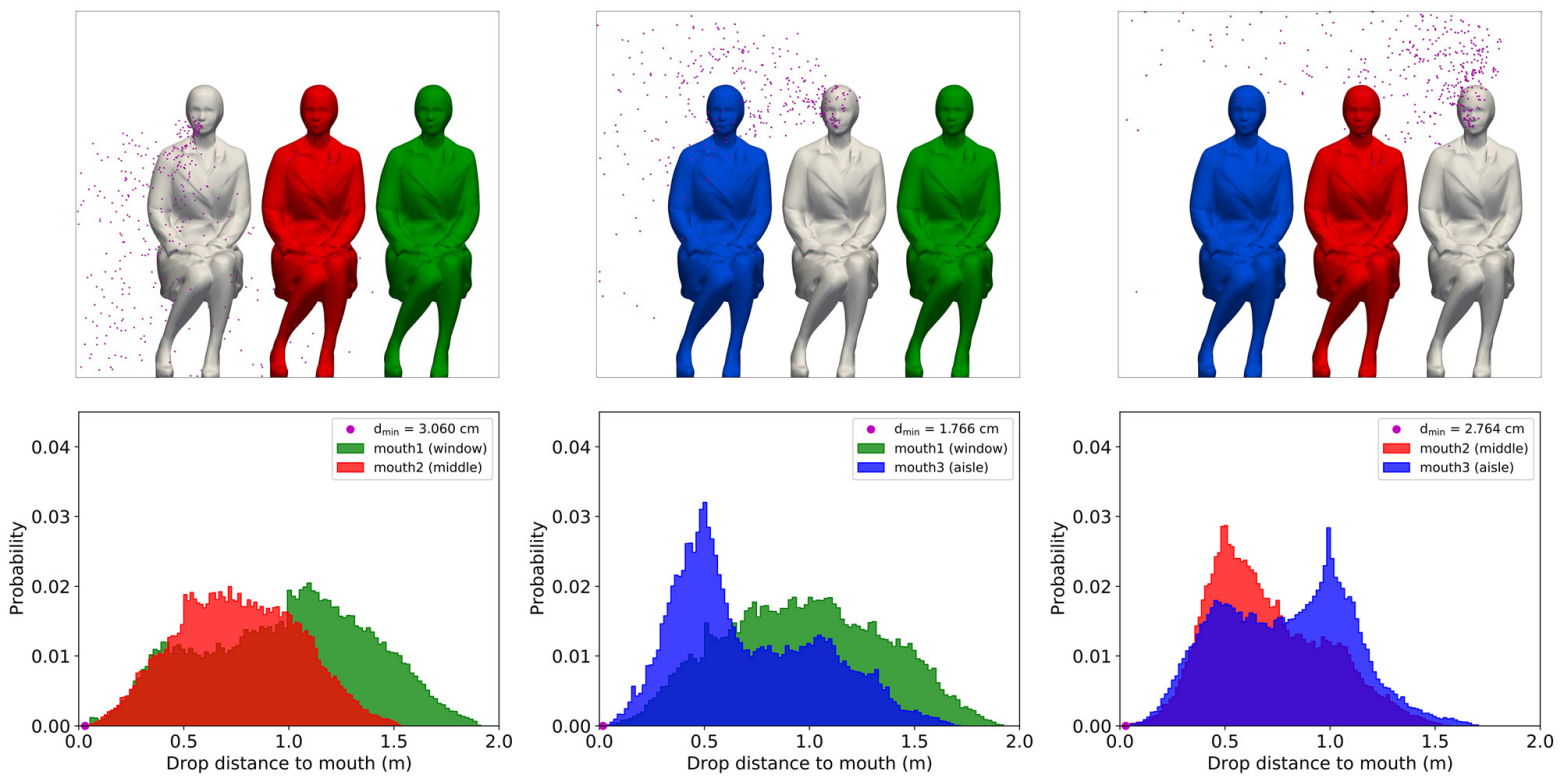
Distance of droplets to mouth histograms calculated during the 40 s of simulation for AISLE (left), MIDDLE (center), and WINDOW (right) scenarios.

Finally, a deeper investigation was performed by analyzing the distance of each drop to the passenger's mouth during its path. Figure 12 shows the probability distribution of the droplets' distance to the mouth for every scenario. The minimum distance was 3.060, 1.766, and 2.764 cm for AISLE, MIDDLE, and WINDOW, respectively. There were in general a small number of droplets passing within a close distance of the mouth. The ventilation system was able to remove an average of 24.1% in the first 40 s from the beginning of the exhalation, reducing the probability of the droplets moving close to the passenger's mouth region.

The system analyzed had a relatively good performance from the perspective of reducing airborne disease transmission risk by focusing on the droplets removed through the exhaust, the mixing and dilution of aerosol concentration, and the distance at which the droplets passed from the mouth. However, it has been noted that there is room for improvement in the design of ventilation systems for reducing the risk of infection. The exhalation mode investigated, with small droplet sizes, was driven by the buoyant flow. Thus, the extraction of aerosols would benefit from a system that facilitates the natural movement of the droplets, and a ventilation system based on an upward flow with an exhaust located at the top could be beneficial for these conditions. Future investigations are recommended to extend this study by combining violent and ordinary respiratory events in a full scale domain, for reproducing drop size distributions representative of the human activity on public transport vehicles. This would contribute to providing a more solid basis for taking mitigation actions and designing and operating future ventilation systems that can create safer environments. Some of the ventilation strategies to investigate are cycles of extraction of particles (displacement, mixed or hybrid ventilation) for reducing the overall mitigation risk from a statistical point of view, or the evaluation of intelligent responsive systems that can dynamically deliver the optimal airflow in a domain.

The results shown in this section are insightful for understanding dynamics of droplets in this kind of domain. In particular, they provided a better understanding of virus spread under asymptomatic transmission conditions of interest for the design of spaces and ventilation systems as well as for optimizing the passenger distribution strategy. It is worth emphasizing that conclusions about risk of infection need to be considered with care and that taking all necessary precautions is recommended to stay safe regardless of the results presented in this work. Infection by contact route may still represent a risk as well as frontal human interaction events. In addition, there are other factors not included in this study that need further consideration in future investigations such as loudness and pitch, head orientation, internal obstacles, seat design, passenger behavior, posture of sitting, or personal characteristics amongst others. In addition, future studies using full scale domain simulations are also advisable to account for the diverse activities in public transport vehicles and remove any bias produced by periodic and symmetry BCs.

## IV. CONCLUSIONS

High spatial and temporal resolution simulations were used for investigating asymptomatic virus transmission in a cross-ventilated space with sitting passengers. A LES coupled with Lagrangian tracking where every drop was represented as an individual entity was modeled. It included transport of carrier species (air and water vapor), multicomponent particle tracking, carrier/particle vapor interaction, inflow synthetic eddy BCs, heat transfer, and thermal radiation. Air injection from the ventilation system was modeled with a multislot diffuser of 105 slots and turbulent inlet conditions for providing a good representation of the injection.

A domain with three sitting female passengers was defined. Dynamics of droplets released from the mouth of the different passengers during the vocalization of vowel /ɑ/ were investigated in detail. For the conditions evaluated, the droplets were initially driven by the exhaled jet and buoyancy and mainly occupied the back region of a hypothetical front row. This effect was more noticeable when droplets were released from positions closer to the ventilation system such as window and middle seat positions. The droplets from the passenger located farthest from the ventilation inlet, such as the one in the aisle, were more affected by the flow from the beginning of the exhalation, and many of the droplets were initially moved to the bottom of the domain. The droplets then encountered primary and secondary flows, which were responsible for the longitudinal and transverse migration. The combination of both these effects made local particle concentrations lower near the mouth or nose regions of other occupants, at the expense of contaminating other rows. Between 19.5% and 27.4% of drops were removed through the outlet during the first 40 s and none of the droplets hit the mouth of the passengers. During their trajectory, the distance of drops to the mouth of the passengers was evaluated, showing that the majority of them passed at a relatively safe distance. However, a few of them passed at a close distance of the order of magnitude of 1 cm.

The results show that further improvements might be applied to cross-ventilation systems for facilitating the natural movement of the exhalation plumes for extracting exhaled droplets. This work presented the results for the vocalization of vowel /ɑ/ representing a single speech-like mode. The generalization of this study to include different speaking events and dynamically accounting for mouth section variations, sound pressure signals, and airflow over time would provide an even more representative scenario. Other factors of oral communication such as loudness and pitch or head orientation need further investigation together with the effect that seat design, passenger behavior, posture of sitting, or personal characteristics have in the overall distribution of droplets.

## Data Availability

The data that support the findings of this study are available from the corresponding author upon reasonable request.
